# Association between parenting practices and children's dietary intake, activity behavior and development of body mass index: the KOALA Birth Cohort Study

**DOI:** 10.1186/1479-5868-8-18

**Published:** 2011-03-14

**Authors:** Jessica S Gubbels, Stef PJ Kremers, Annette Stafleu, Sanne I de Vries, R Alexandra Goldbohm, Pieter C Dagnelie, Nanne K de Vries, Stef van Buuren, Carel Thijs

**Affiliations:** 1 NUTRIM School for Nutrition, Toxicology and Metabolism, Department of Health Promotion, Maastricht University Medical Centre, PO Box 616, 6200 MD Maastricht, the Netherlands; 2 Department of Biosciences, TNO Quality of Life, PO Box 360, 3700 AJ Zeist, the Netherlands; 3 Department of Prevention & Health, TNO Quality of Life, PO Box 2215, 2301 CE Leiden, the Netherlands; 4 School for Public Health and Primary Care (CAPHRI), Department of Epidemiology, Maastricht University Medical Centre, PO Box 616, 6200 MD Maastricht, the Netherlands; 5 Department of Statistics, TNO Quality of Life, PO Box 2215, 2301 CE Leiden, the Netherlands; 6 Department of Methodology and Statistics, FSW, University of Utrecht, PO Box 80125, 3508 TC Utrecht, the Netherlands

## Abstract

**Background:**

Insights into the effects of energy balance-related parenting practices on children's diet and activity behavior at an early age is warranted to determine which practices should be recommended and to whom. The purpose of this study was to examine child and parent background correlates of energy balance-related parenting practices at age 5, as well as the associations of these practices with children's diet, activity behavior, and body mass index (BMI) development.

**Methods:**

Questionnaire data originated from the KOALA Birth Cohort Study for ages 5 (N = 2026) and 7 (N = 1819). Linear regression analyses were used to examine the association of child and parent background characteristics with parenting practices (i.e., diet- and activity-related restriction, monitoring and stimulation), and to examine the associations between these parenting practices and children's diet (in terms of energy intake, dietary fiber intake, and added sugar intake) and activity behavior (i.e., physical activity and sedentary time) at age 5, as well as BMI development from age 5 to age 7. Moderation analyses were used to examine whether the associations between the parenting practices and child behavior depended on child characteristics.

**Results:**

Several child and parent background characteristics were associated with the parenting practices. Dietary monitoring, stimulation of healthy intake and stimulation of physical activity were associated with desirable energy balance-related behaviors (i.e., dietary intake and/or activity behavior) and desirable BMI development, whereas restriction of sedentary time showed associations with undesirable behaviors and BMI development. Child eating style and weight status, but not child gender or activity style, moderated the associations between parenting practices and behavior. Dietary restriction and monitoring showed weaker, or even undesirable associations for children with a deviant eating style, whereas these practices showed associations with desirable behavior for normal eaters. By contrast, stimulation to eat healthy worked particularly well for children with a deviant eating style or a high BMI.

**Conclusion:**

Although most energy balance-related parenting practices were associated with desirable behaviors, some practices showed associations with undesirable child behavior and weight outcomes. Only parental stimulation showed desirable associations with regard to both diet and activity behavior. The interaction between parenting and child characteristics in the association with behavior calls for parenting that is tailored to the individual child.

## Background

Eating and physical activity (PA) habits originate in early childhood [1,2], and track into later life [3,4]. Parents can have a strong influence on their children's dietary intake and activity behavior: they can control the availability of and exposure to food and activity opportunities, they act as role models, provide their children with support and structure, and use specific parenting practices [5]. In contrast to the overall parenting style, which refers to general patterns of parenting and the emotional climate in which parents' behaviors are expressed, parenting practices are content-specific acts of parenting [6], such as rules about dietary intake or activity behavior. The current study focused on the latter, i.e., behavior-specific parenting practices.

Many studies have examined the influence of food-related parenting practices and feeding styles on children's dietary intake and weight. Restricting the intake of unhealthy food items, for instance, has been found to be associated with a higher intake of those items and with a higher body mass index (BMI; see e.g., the following reviews [7-9]). Other studies, however, have found associations between restriction and desirable dietary intake behavior [e.g., [10,11]]. Studies examining diet-related parenting practices other than restriction have also reported inconsistent results [7]. Promotion, stimulation or pressure to eat certain foods have been reported to have both favorable [e.g., [12-14]] and unfavorable [e.g., [15]] effects on children's diet, and were found to be associated with a lower child BMI [e.g., [16]]. Conflicting findings have also been found with regard to monitoring children's dietary intake, which was reported to be associated with childhood overweight [e.g., [17]], but also with a lower child BMI [e.g., [18]] and a healthier diet [19]. Many of these studies used a cross-sectional design. For example, 19 of the 22 studies included in the review by Faith and colleagues [7] on the effects of feeding strategies were cross-sectional, rather than longitudinal. The results of cross-sectional studies are difficult to interpret, which might explain the conflicting findings reported by these studies. These conflicting findings also led us to the decision not to formulate specific hypotheses for the current study regarding the directions of the associations between diet-related parenting practices and children's dietary intake and BMI. The parenting practices examined in the current study were restriction of unhealthy intake, monitoring a child's diet and stimulation of healthy intake.

As regards activity behaviors, many studies have examined the association between parental support and encouragement to be physically active, which seem to be important positive predictors of children's PA [20]. Many other studies, however, did not find an effect on PA [21]. Explicit rules restricting television watching have been found to be associated with less sedentary behavior [e.g., [22-24]], but also with lower levels of PA in boys and higher levels of PA in girls [e.g., [22]]. Monitoring a child's activity has been found to be associated with increased PA [19]. The activity-related parenting practices examined in the current study were restriction of sedentary time, monitoring a child's activity behavior and stimulation of PA. As with diet-related practices, we did not formulate specific hypotheses regarding the directions of the associations between activity-related parenting practices and children's activity behavior and BMI.

Examining the effect of different parenting practices on energy balance-related behaviors is important to ascertain which practices should be recommended to parents to prevent childhood obesity. In addition, it is important to assess to whom these practices should be recommended, implying research into the association between background characteristics and parenting practices. For example, the use of more controlling diet-related parenting practices, including more restriction, has been shown to be associated with several parental characteristics, including lower BMI, higher educational level and social class, both older and younger age, white ethnicity and employment [10,19,25-28]. Pressure to eat was found to be positively related to parental non-white ethnicity, female gender, and lower socioeconomic status, and diet monitoring to maternal older age, higher BMI and higher educational level [26,29,30]. It is not only parental characteristics which appear to be related to specific parenting practices: child characteristics have also been found to evoke different parenting practices. For example, controlling practices, encouragement or pressure, and monitoring have all been found to correlate with either higher or lower child weight [e.g., [30-33]], and controlling practices were used more for girls than for boys [34]. It is less clear which background factors predict the use of activity-related parenting practices, although a study among a Latino sample showed that parental employment is associated with more control over the child's PA [19]. Based on these previous findings, we hypothesized that the following child characteristics would be associated with the use of various energy balance-related parenting practices: weight status-related variables (i.e., the child's birth weight and BMI [e.g., [30-33]]) and gender [34]. We also hypothesized that children's eating style and activity style would be associated with parenting practices. As regards parental background characteristics, we expected the following variables to be associated with the parenting practices: parental BMI [26-29], educational level [10,26,30], employment [19,26], ethnicity (i.e., country of birth) [26], and age [10,19,26,27,29].

It has been claimed that there is an urgent need to know whether the effects of parenting practices are similar across different groups of children [e.g., [10,18]]. Answering this question requires moderation research [35]. Several child characteristics may moderate the effects of diet-related parenting practices. Dietary restriction and control have been reported to have stronger undesirable effects on the dietary intake of girls than of boys [e.g., [7,19]], but studies regarding the moderation of gender in the relationship between restriction and weight status have reported mixed results [7]. Recently, we reported that restriction showed an association with desirable dietary intake for normal weight children, but not for overweight children [10]. Also, the association between restriction and desirable dietary intake behavior of 2-year-olds was found to be weaker or even absent in children with a problematic eating style (i.e., those who do not like many foods, eat reluctantly, or are slow eaters [10]). Again, empirical evidence regarding moderators of parental influences on child activity behavior is generally lacking, but in line with findings regarding the dietary intake domain, we hypothesized that similar interactions between parenting practices and child factors would exist for the activity domain. Based on the studies referred to above, we hypothesized that the following child characteristics would moderate the associations between parenting practices and child energy balance-related behavior and BMI: weight-related variables (i.e., birth weight and BMI) [10], gender [e.g., [7,19]], and eating style and activity style [10].

**Figure 1:**
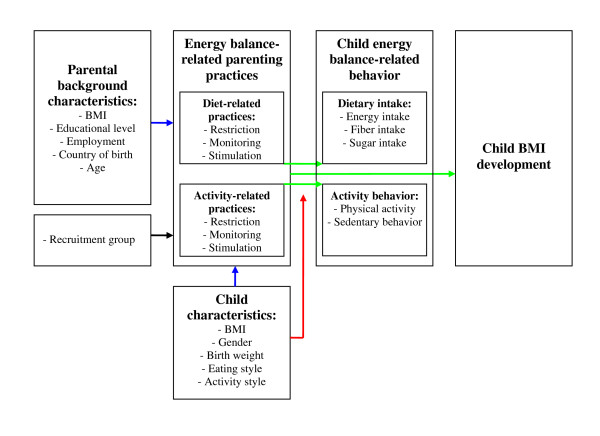
**Model of energy balance-related parenting**.

Figure 1 shows a summary of our main hypotheses. The present study examined parental and child associates of energy balance-related (i.e., diet-related and activity-related) parenting practices (blue arrows in Figure 1). We also examined the association between energy balance-related parenting and activity behavior and dietary intake in 5-year-old children, as well as the prospective influence of these practices on children's BMI development up to age 7 (green arrows). Finally, based on previous studies [e.g., [10,18]], we examined whether child background characteristics moderated the impact of the parenting practices (red arrow).

## Methods

### Respondents and procedure

The KOALA Birth Cohort Study (the Netherlands) is a prospective cohort study which started in the year 2000. Healthy pregnant women were recruited from an existing cohort for a study of the etiology of pregnancy-related pelvic girdle pain, as well as through recruitment channels from among 'alternative lifestyle' circles (e.g., through anthroposophist midwives and general practitioners, and organic food shops [36]). The latter group of women (17.9%) were likely to have an alternative lifestyle in terms of dietary habits (e.g., preferring organic foods), child rearing, vaccination programs, antibiotics use, etc. All participants signed informed consent, and approval was obtained from the Maastricht University/University Hospital Maastricht medical ethics committee. In total, the mothers of 2834 children participated and completed mail-based questionnaires during pregnancy and regularly after birth. Ten children were excluded because of congenital defects (e.g., Down syndrome).

### Questionnaires

When the children were around 5 years old, parents completed a questionnaire regarding their energy balance-related parenting practices, their child's dietary intake, activity behavior, weight and height, and several other child and parent characteristics. A total of 2026 questionnaires (71.7%) were returned. Children for whom the 5-year questionnaire was returned had a slightly higher birth weight compared to children for whom this questionnaire was not returned (3521 vs. 3468 grams, p < 0.05). At age 7, a follow-up questionnaire was sent, assessing only child weight and height, and questionnaires regarding 1819 (89.8%) children were returned. There was no selective attrition between age 5 and age 7 with regard to BMI z-score at age 5 (p > 0.05).

#### Child background characteristics

The child's eating style was assessed on two dimensions: the child's picky eating [37] and the child's appetite. We also assessed whether the child had an active activity style. For more information about these concepts, see Table 1. In addition, the child's birth weight (in grams) and gender were also assessed in the current study.

**Table 1:** Descriptive and scale information of child characteristics and parenting practices (N = 2021)

Category		Concept		Item(s)	**Answering scale**^a^	**Reliability**^b^	Mean (SD)
**Child characteristics**	*Diet*	Eating style	Hungry	Compared to peers, my child is always hungry.	A	-	2.65 (0.85)
			Picky (CFQ)	- My child's diet conists of only a few foods. - My child is unwilling to eat many of the foods I serve. - My child is picky or fussy about what (s)he eats.	A	0.74	2.27 (1.05)
	
	*Activity*	Activity style	Active	Compared to peers, my child.. - ..is very active. - ..never sits still.	A	0.76	3.36 (0.88)

**Parenting practices**	*Diet*	Restriction unhealthy intake (CFQ)		- I have to be sure that my child does not eat.. * ..too many sweets (candy, ice cream, pastries). * ..too many high-fat foods. * ..too much of his/her favorite foods. - As a reward for good behavior, I offer my child.. * ..his/her favorite foods. * ..sweets (candy, ice cream, pastries). - If I did not guide or regulate my child's eating, (s)he would eat.. * ..too much of his/her favorite foods. * ..too many junk foods. - I intentionally keep some foods out of my child's reach.	A	0.63	3.32 (0.61)
		
		Monitoring intake (CFQ)		How much do you keep track of.. - ..the sweets (candy, ice cream, pies, pastries) that your child eats? - ..the snack food (potato chips, nuts, cheese puffs) that your child eats? - ..the high-fat foods that your child eats?	B	0.86	4.41 (0.56)
		
		Stimulation healthy intake		- I make sure that my child eats enough healthy food products. - I get my child enthusiastic about healthy products, such as vegetables, fruit and whole meal products.	A	0.54	4.57 (0.55)

**Parenting practices**	*Activity*	Restriction sedentary behavior		- I have to be sure that my child does not.. * ..watch too much television. * ..play too many computer games. - As a reward for good behavior, I put on a nice video/DVD/computer game for my child. - If I did not guide or regulate my child's activity behavior, (s)he would.. * watch too much television or play too many computer games. * not get enough physical activity. - I intentionally keep my child away from the television or computer.	A	0.59	3.01 (0.68)
	
		Monitoring activity		How much do you keep track of... - ..the amount of television your child watches and how many computer games (s)he plays? - ..the amount of physical activity your child has?	B	0.65	3.94 (0.78)
		
		Stimulation to be active		- If my child says "I don't feel like walking or bicycling to there", I try to get him/her to do this anyway. - I have to be careful that my child gets enough exercise. - I make sure that my child travels actively on foot or by bicycle (with or without me) as often as possible.	A	0.57	4.26 (0.65)

^a ^Answering scale: A. 1 *(completely disagree) *to 5 *(completely agree)*; B. 1 *(never) *to 5 *(always)*

^b ^Reliability of the sum scale as measured by Cronbach's α.

Notes: SD = standard deviation, CFQ = Child Feeding Questionnaire [37].

#### Parental background characteristics

The questionnaire assessed the number of working hours per week of the father and mother, their educational level and their country of birth. Educational level was recoded into three levels (low, medium and high), in line with international classification systems [38]. Country of birth was recoded into 'Netherlands' versus 'other'. Maternal age at the time of the child's birth was also assessed in the current study.

#### Parenting practices

We assessed parenting practices regarding children's dietary intake and activity behavior. The items used to assess these parenting practices and the corresponding Cronbach's α values are listed in Table 1. The parenting practices 'restriction' of unhealthy intake and 'monitoring' were assessed using the validated scales of the Child Feeding Questionnaire (CFQ [37]), translated into Dutch. Since our study focused on parenting practices in relation to weight gain prevention, we considered 'stimulation of healthy intake' to be more suitable than the original 'pressure' to eat scale of the CFQ. Pressure to eat is a practice which is often used to increase children's weight [39]. In addition, we 'converted' the diet-related items of the CFQ to the activity context in order to create an 'Activity-related Parenting Questionnaire', consisting of three scales similar to the diet-related CFQ scales: 'restriction of sedentary behavior', 'monitoring activity' and 'stimulation to be physically active'. The similarity between the diet-related scales and the activity-related scales enabled cross-behavioral comparison of the correlates and the effects of the energy balance-related parenting practices. The 'Activity-related Parenting Questionnaire' has, however, not been previously validated.

#### Child dietary intake, activity behavior and BMI

Children's dietary intake was assessed using a Food Frequency Questionnaire (FFQ), assessing intake during the 4 weeks preceding the questionnaire. This FFQ was specifically developed to assess children's energy intake, and was validated using the doubly labeled water method [40]. The FFQ consisted of 71 items. Additional questions were asked for 27 foods, asking for the specific types or brands consumed and preparation methods. Parents indicated their child's habitual consumption frequency of each of the food items by checking 1 of 6 frequency categories ranging from 'never' to '6-7 days a week'. Respondents were asked to report portion sizes in natural units (e.g., pieces, slices), household units (e.g., glasses, spoons) or grams (e.g., grams of meat). Parents were asked to measure the volume of the cups and glasses they used for the children. The average energy intake (kJ) and fiber intake (in grams per MJ) per day were calculated using the 2001 Netherlands Food Composition (NEVO) table [41]. Nutritional values of products that were not (or not yet) included in the 2001 NEVO table were provided by a dietician. Added sugar intake (expressed as a percentage of total energy intake), which is not included in the NEVO table, was calculated using values from an earlier study [42]. Added sugar was defined as the amount of saccharose, glucose and/or fructose added to a food or meal by the consumer or the manufacturer.

Children's activity behavior was assessed using questions based on a standard questionnaire for measuring activity behavior, used in Dutch Youth Health Care [43]. Parents were asked on how many days in a normal week during the last 4 weeks their child had gone to school on foot or by bicycle, had played sports at school (e.g., during physical education lessons), had played sports outside the school at a sports club, and had played outside (outside school hours). A second item assessed the average duration of each of these activities. The duration and number of days were multiplied to calculate the number of minutes spent on a particular activity per week. The number of minutes spent on the various activities were then added up to calculate the total number of minutes of physical activity per week, which was divided by 7 to get the average time (in minutes) the children were physically active per day. Sedentary screen-based behavior was assessed in a similar manner, asking parents about their child's television watching (including videos and DVDs) and computer playing.

In the 5-year questionnaire, parents were asked to report their child's weight and height (measured without shoes and clothes, specified to one decimal), in order to calculate the child's body mass index (BMI, i.e., weight (kg)/(height (m))^2^). BMI was then recoded into BMI z-scores compared to the 1997 national reference population (i.e., the Fourth Dutch National Growth Study [44]).

At age 7, parents were asked to report their child's height and weight again, and these values were recoded into BMI z-scores for the age of 7 years.

### Data analyses

The analyses were conducted using SPSS 15.0. Cronbach's α values were calculated as an estimate of the lower bound of reliability of the scales used (e.g., the CFQ scales). All analyses described below were adjusted for recruitment group (alternative versus conventional lifestyle), and p-values < 0.05 were considered statistically significant.

First, linear regression analyses were used to examine child and parent background correlates of the use of the six energy balance-related parenting practices (i.e., restriction of intake, monitoring intake, stimulation of healthy intake, restriction of sedentary time, monitoring activity, and stimulation to be active). This is illustrated by the blue arrows in Figure 1. The correlates that were examined were child background characteristics (child gender, birth weight, BMI z-score at age 5, activity style, hungry eating style and picky eating style) and parental background characteristics (parental BMI, employment, educational level, and country of birth; maternal age). All correlates were entered simultaneously, correcting for potential confounding by the other variables.

Second, linear regression models were fitted to assess the associations between the parenting practices and each of the six outcome variables: energy intake, fiber intake, added sugar intake, PA, and sedentary behavior (all at age 5), and BMI z-score at age 7 (see Figure 1, green arrows). The diet-related parenting practices were included in the analyses using the dietary intake variables as the outcome, while the activity-related parenting practices were included in the analyses using PA and sedentary time as an outcome. Both diet-related and activity-related parenting practices were included in the analyses examining the influence on BMI at age 7. These analyses were adjusted for the child characteristics and parental background characteristics described above, including child BMI z-score at age 5. The analyses with BMI z-score at age 7 as a dependent variable thus reflected BMI z-score development between ages 5 and 7. The analyses with the BMI z-score at age 7 as the dependent variable were repeated after excluding children who were underweight (BMI z-score < 5^th ^percentile) at age 5, to examine whether this affected the findings.

Third, in order to examine whether child characteristics moderated the association between parenting practices and children's energy balance-related behavior and BMI development, we calculated interaction terms between the parenting practices and the various child characteristics (i.e., child gender, birth weight, BMI z-score at age 5, activity style, hungry eating style and picky eating style; see Figure 1, red arrow). The interaction terms were added to the regression analyses described above (i.e., the model including the parenting practices, adjusted for the parent and child background characteristics) in a separate step using a stepwise forward entering procedure [45]. This forward procedure involved adding the interaction term that had the highest correlation with the unexplained variance of the outcome variable to the model, on condition that it significantly improved the predictive value of the model. This procedure was repeated until the predictive value of the model could no longer be significantly improved by any of the interaction terms not yet included in the model [45]. Subsequently, stratified linear regression analyses were performed for the interaction terms that were included in the model in this separate step, in order to examine the association with the parenting practice in the different strata of the moderator variable (i.e., the child characteristic). Continuous variables (e.g., child birth weight) were dichotomized for this purpose, using a median split. We only report the interactions for which the association between the parenting practice and the outcome was statistically significant in either or both of the strata of the moderator variable.

## Results

Of the 2026 children participating in the questionnaire survey around age 5, 51.2% were male. The children's mean daily energy intake was 6176 kJ (1467 kCal), with a standard deviation (SD) of 1286 kJ (306 kCal). The children consumed an average of 2.5 grams of dietary fiber per MJ of energy intake (SD = 0.6), while added sugar intake contributed 15.8% (SD = 6.6%) to their total energy intake. Children were physically active for an average of 116 minutes per day (SD = 55), and spent 59 minutes on sedentary screen-based activities (SD = 42). Mean BMI z-score at age 5 was -0.27 (SD = 0.99), compared to -0.29 (SD = 0.94) at age 7. Descriptive information regarding children's eating styles and activity style is listed in Table 1. Mean maternal BMI was 24.0 kg/m^2 ^(SD = 3.8), mean paternal BMI was 25.0 kg/m^2 ^(SD = 3.1). Mothers worked an average of 18.0 hours (SD = 11.1) and fathers 37.8 hours (SD = 10.1) per week. A total of 3.0% of the mothers and 3.7% of the fathers had not been born in the Netherlands. Educational level was high for 54.1% of the mothers and 53.2% of the fathers, medium for 38.0% and 33.9%, and low for 7.9% and 12.9%, respectively. Average maternal age at the time of the birth of their child was 32.2 years (SD = 3.7).

### Correlates of parenting practices

Several child characteristics were significantly related to the parenting practices used at age 5 years (Table 2). Parents imposed more dietary restriction on girls than on boys. The reverse was true for restriction of sedentary time: boys were more restricted by their parents than girls. A higher child BMI z-score (at age 5) was associated with more dietary restriction and more stimulation of healthy intake. Dietary intake was more restricted by parents whose child had a hungry or picky eating style. Children who were picky eaters were less likely to be stimulated to eat healthy. Children with an active activity style were less likely to be restricted in their sedentary time, but also more likely to be stimulated to be physically active than their normal peers.

**Table 2:** Child and parent correlates of parenting practices at child's age of 5 years (N = 2021)

				Standardized regression coefficients (β)^*a*^					
				**Parenting Practices**					
				***Diet-related***			***Activity-related***		
				**Restric-tion un-healthy**	**Monito-ring**	**Stimu-lation healthy**	**Restric-tion se-dentary**	**Monito-ring**	**Stimu-lation PA**

**Child characteristics**	*General*	Gender *(boy = 1, girl = 2)*		0.08**	0.01	0.03	-0.07**	0.00	-0.02

		Birth weight *(grams)*		0.00	0.00	-0.03	0.03	0.01	0.00
		BMI z-score 5 years		0.10***	0.05	0.07*	0.05	0.03	0.03
	
	*Diet*	Eating style	Hungry	0.14***	-0.05	-0.04	^*b*^	^*b*^	^*b*^
			Picky	0.21***	0.00	-0.21***	^*b*^	^*b*^	^*b*^
	
	*Activity*	Activity style	Active	^*b*^	^*b*^	^*b*^	-0.13***	-0.02	0.06*

**Parental characteristics**	*Maternal*	BMI		-0.10**	-0.03	-0.14***	-0.05	-0.03	-0.05
		Educational level *(compared to medium)*	Low	-0.05	-0.02	-0.07*	0.02	0.00	-0.07*
			High	0.01	0.03	0.01	0.09**	0.04	0.02
		Employment *(hours per week)*		-0.01	-0.07*	-0.03	0.00	-0.10***	-0.09**
		Country of birth *(NL = 0, other = 1)*		-0.01	-0.02	-0.03	-0.01	-0.01	0.00
		Age at birth of child *(years)*		0.00	-0.01	0.03	0.01	-0.02	-0.03
	
	*Paternal*	BMI		0,01	-0.02	0.02	0.03	-0.03	0.03
		Educational level *(compared to medium)*	Low	0.00	-0.02	0.01	-0.05	-0.04	0.02
			High	0.04	-0.07*	0.03	0.02	0.03	-0.02
		Employment *(hours per week)*		0.00	0.01	-0.03	-0.01	0.03	0.00
		Country of birth *(NL = 0, other = 1)*		0.05	0.02	0.00	-0.01	0.01	-0.01

* p < 0.05; ** p < 0.01; ***p < 0.001

^*a *^Parenting practices were assessed on a scale from 1-5. All independent variables were entered simultaneously. All analyses were adjusted for recruitment group.

^*b *^Variable not included in the current analysis. Eating style was only included as a predictor in the analyses using diet-related practices as dependent variables; activity style was only included in the analyses using activity-related practices as dependent variables.

Notes: BMI = Body mass index, PA = Physical activity, NL = the Netherlands.

Various maternal characteristics were associated with the parenting practices (Table 2). A higher maternal BMI was associated with less restriction and less stimulation with regard to dietary intake. Maternal educational level was positively associated with stimulation of healthy intake, stimulation to be physically active and restriction of sedentary time, while mothers' working hours were negatively related to the monitoring of both dietary intake and activity behavior, and to stimulation to be physically active. Paternal educational level was inversely associated with monitoring of dietary intake.

### Associations between parenting practices and diet and activity behavior at age 5

Stimulation of healthy intake and monitoring a child's diet were not only associated with more dietary fiber intake, but also with less added sugar intake (Table 3). Children's energy intake was not associated with the parenting practices. Restriction of sedentary behavior was related to more sedentary time and less PA. Stimulation to be active was positively associated with PA, and negatively with sedentary behavior.

**Table 3:** Association between parenting practices at child's age 5 and child's dietary intake and activity behavior at 5 years (N = 2021)

		Standardized regression coefficients (β)^*a*^				
Parenting practice		Energy intake *(kJ/day)*	Fiber intake *(g/MJ)*	Added sugar intake *(En%)*	Physical activity *(minutes/day)*	Sedentary behavior *(minutes/day)*
**Diet**	*Restriction unhealthy intake*	-0.03	-0.01	-0.04	^*b*^	^*b*^
	*Monitoring intake*	-0.02	0.09**	-0.08**	^*b*^	^*b*^
	*Stimulation healthy intake*	0.01	0.18***	-0.07**	^*b*^	^*b*^

**PA**	*Restriction sedentary time*	^*b*^	^*b*^	^*b*^	-0.19***	0.09**
	*Monitoring activity*	^*b*^	^*b*^	^*b*^	0.03	-0.03
	*Stimulation PA*	^*b*^	^*b*^	^*b*^	0.12***	-0.12***

** p < 0.01; ***p < 0.001

^*a *^Analyses were adjusted for child BMI z-score at age 5, recruitment group (alternative vs. conventional), child background characteristics (gender, birth weight, activity style and eating style) and parent background characteristics (parental BMI, educational level, employment and country of birth; and maternal age).

^*b *^Variable not included in the current analysis. Diet-related parenting practices were only included as a predictor in the analyses using dietary behaviors as dependent variables; activity-related parenting practices were only included in the analyses using the activity behaviors as dependent variables.

Notes: kJ = kilo Joules; g/MJ = intake in grams of nutrient per MJ of total energy intake; En% = Energy intake of nutrient as a percentage of total energy intake; PA = Physical activity.

### Associations between parenting practices and BMI development from age 5 to age 7

All analyses regarding BMI at age 7 were corrected for BMI at age 5, so that BMI results at age 7 reflect BMI development from 5 to 7 years of age. Stimulation of healthy intake was negatively associated with BMI development up to age 7, while restriction of sedentary time was positively related to BMI development (Table 4). Repeating the analyses while excluding children who were underweight at age 5 (N = 187) did not substantially change the results.

**Table 4:** Association between parenting practices at age 5 and child's BMI development up to age 7

		Standardized regression coefficients (β)^*a*^	
Parenting practice		BMI z-score *(including underweight^b ^children, N = 1513)*	BMI z-score *(excluding underweight ^b ^children, N = 1326)*
**Diet**	*Restriction unhealthy intake*	0.01	0.01
	*Monitoring intake*	0.02	0.03
	*Stimulation healthy intake*	-0.06*	-0.07*

**Activity**	*Restriction sedentary time*	0.06*	**0.05^†^**
	*Monitoring activity*	-0.02	0.00
	*Stimulation PA*	0.01	-0.01

^†^p < 0.10; * p < 0.05

^*a *^Analyses were adjusted for child BMI z-score at age 5, recruitment group (alternative vs. conventional), child background characteristics (gender, birth weight, activity style and eating style) and parent background characteristics (parental BMI, educational level, employment and country of birth; and maternal age).

^*b *^Underweight at age 5 was defined as a BMI z-score < 5^th ^percentile.

Notes: BMI = Body mass index, PA = Physical activity.

### Child background characteristics as moderators of parenting practice impact

**Figure 2:**
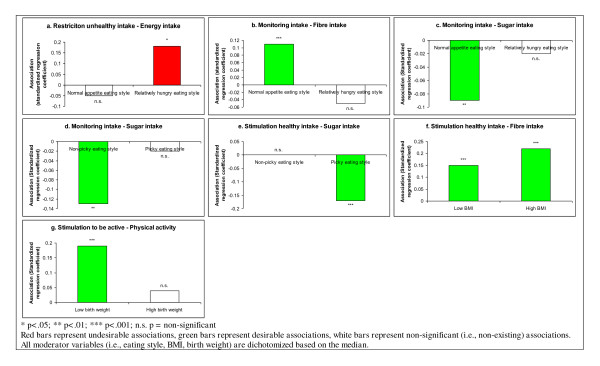
**Significant child characteristics moderating the association between parenting practices and behaviour**.

There were several significant interactions between parenting practices and child background characteristics, an overview of which is provided in Figure 2. The green bars in Figure 2 represent associations between parenting practices and desirable behavior (i.e., increased healthy intake/decreased unhealthy intake as regards diet-related practices; increased PA/decreased sedentary time as regards activity-related practices), while the red bars represent associations with undesirable behavior (i.e., decreased healthy intake/increased unhealthy intake; decreased PA/increased sedentary time).

The associations between the diet-related practices and children's dietary intake were found to be moderated by the children's eating style and weight status. Restriction was associated with increased energy intake for children who were characterized as relatively hungry (standardized β = 0.18, p < 0.05), but not for their peers with a normal appetite (β = -0.05, non-significant (n.s.); see Figure 2a). The desirable associations between diet monitoring and both dietary fiber and added sugar intake were not found for hungry children or picky eaters (absolute β values < 0.05, n.s.). These desirable associations were, however, found for children who were not reported to be relatively hungry (β = 0.11, p < 0.001 for fiber intake; β = -0.09, p < 0.01 for sugar intake; see Figures 2b and 2c), and were not reported to be picky eaters (β = -0.13, p < 0.01 for sugar intake; Figure 2d). By contrast, the desirable association between stimulation of healthy intake and added sugar intake was found for picky eaters (β = -0.17, p < 0.001), but not for normal, non-picky eaters (β < 0.01, n.s.; Figure 2e). Stimulation of healthy intake showed a slightly stronger desirable association with fiber intake for children with a BMI above the median at age 5 (β = 0.22, p < 0.001), compared to children with a lower BMI at age 5 (β = 0.15, p < 0.001; Figure 2f). There were no interactions between diet-related practices and the child's gender.

Regarding activity behavior, the desirable association between stimulation to be physically active and the child's PA was only found for children with birth weight below the median (β = 0.19, p < 0.001), and not for children with a higher birth weight (β = 0.04, n.s.; see Figure 2g). There were no interactions between activity-related practices and the child's gender or activity style.

## Discussion

The current study examined child and parent correlates of energy balance-related parenting practices, as well as the association between these practices and diet and activity behavior at age 5, and BMI development from age 5 to 7 years. Parents were found to be more restrictive regarding their daughters' diet than their sons', which is in line with previous research [34]. However, the current study also showed that girls were less restricted than boys when it came to sedentary time. Parents may have different priorities for boys and girls when it comes to restricting unhealthy behaviors; perhaps inactivity is of greater concern to parents where their sons are concerned, while overconsumption is of greater concern to parents where their daughters are concerned. Parental restriction of unhealthy intake was also positively associated with child BMI, in agreement with previous studies [31]. Child BMI was also positively associated with parental stimulation of healthy intake. Both the increased restriction of unhealthy intake and the increased stimulation of healthy intake in heavier children might reflect reactions of parents to their child's weight, trying to get heavier children to eat a healthier diet so as to decrease their weight. A similar mechanism might be operative for children with a hungry or picky eating style, who were shown to be more restricted by their parents. Parents might feel that these children need more external control over their eating to compensate for their deviant eating style. In view of the cross-sectional nature of our data, however, we cannot exclude the possibility that these children's eating style actually became more deviant in reaction to the strict control their parents exercised over their diet. In line with the latter explanation, various studies have reported that high parental control over child eating interferes with children's self-control over their intake [e.g., [9]], thus leading to a deviant eating style.

There were also several parental characteristics that predicted which practices parents would apply. Maternal BMI was found to be inversely associated with dietary restriction and stimulation, which confirms previous findings [26-28]. Maternal educational level was positively associated with stimulation of both healthy intake and PA. This adds to previous research showing that parental education is positively associated with restriction and other controlling practices [10,25,26]. The number of hours that the mothers worked was negatively associated with monitoring their children's diet and activity behavior and stimulation to be physically active. A similar association was previously reported by Brown and colleagues [25], showing that parents who stayed at home to take care of their children exercised stricter control over their children's diet. As working parents leave part of the child rearing to others, such as child-care staff [e.g., [46]], they may be inclined to be less strict during the limited time they can spend with their children.

With regard to the associations between parenting practices and their children's behavior and BMI development, we found that monitoring a child's diet and stimulating healthy intake were both associated with the child having a healthy diet. Stimulation of healthy intake even had a desirable effect on the child's BMI development up to the follow-up at age 7. By contrast, dietary restriction was not associated with any of the dietary outcomes, nor was it associated with BMI development. Previous studies have shown conflicting results with regard to all three of the above parenting practices (monitoring [e.g., [17-19]]; stimulation [e.g., [12-15]]; restriction [e.g., [7-11]]), with some studies supporting our findings and some contradicting them. We believe that the key to resolving these conflicting findings might lie in the interaction between children and parents. In line with our hypotheses based on previous studies [e.g., [10,18]], the current study showed that the associations between parenting practices and child behavior and weight development depended on the children's characteristics. Dietary restriction was associated with undesirable dietary intake behaviors by children with a deviant eating style (i.e., children who were relatively hungry compared to peers). In line with this, previous research showed that the associations between restriction and desirable dietary intake behavior at a very young age (2 years) were partly lacking in children with deviant eating styles [10]. Analogous to our findings with regard to restriction, the associations between monitoring and a desirable child diet were not found for relatively hungry children or picky eaters. By contrast, stimulation to eat healthy was found to be specifically beneficial for picky eaters, as well as for children with a high BMI. In line with previously raised hypotheses [10], this indicates that although restriction and monitoring might be less suitable for children with certain unfavorable characteristics (e.g., deviant eating style, high BMI), stimulating these children to eat a healthy diet seems all the more effective for them. It is worrying, however, that picky eating also correlated with less parental stimulation to eat healthy. Educating parents might therefore be an important step toward improving children's diet, perhaps especially for children with a deviant eating style.

The effects of rules about television viewing on activity behaviors have previously been found to depend on the child's gender, with desirable effects on girls, but undesirable effects on boys [22]. We did not find indications of such a difference in the current study, but we did find undesirable correlations between restriction of sedentary time and behavior and BMI development, for both boys and girls; restriction was associated with increased sedentary time, decreased PA, and an increased BMI development up to age 7. This contradicts previous studies that showed that explicit rules restricting children's television watching were associated with less viewing time [22-24]. An explanation for these contradictory findings might lie in the assessment of restriction of sedentary time in the current study, which not only included explicit rules limiting television and computer use, as in the previous studies, but took a broader view of restrictive parenting. For example, the measure of restriction of sedentary behavior in the current study included items assessing what parents thought would happen if they did not restrict their child's sedentary behavior (see Table 1). The inclusion of such broader items was based on the diet-related restriction scale of the CFQ [37]. Stimulation to be active was positively associated with children's PA and negatively with sedentary time in our study, which is in line with a review showing that encouragement and support are important predictors of increased PA [20].

The findings of the current study have implications for both research and practice. With regard to research, studies into the effects of parenting practices that do not incorporate the possibility of moderation by child characteristics will tend to produce conclusions on the effects of parenting practices that strongly depend on their study population. In addition to the moderators identified in the current study, previous research has revealed several additional child factors that moderate the effects of parenting practices, including the child's personality, temperament [10,11] and gender [7]. These interactions might also contribute to the many contradictions in the current evidence base on diet-related parenting practices. Therefore, we believe that research into the effects of parenting practices cannot be limited to the direct association between practices and outcomes, but should always incorporate a theory-based examination of possible moderation effects [35,47]. The practical implications of the current findings are that overweight prevention interventions targeted at parenting practices should be tailored to individual child characteristics, since specific parenting practices might be beneficial for one child, but useless (or even potentially disadvantageous) for another.

The current study had several strengths and limitations. One of the strengths is that it included a longitudinal follow-up to assess the effects on BMI development. However, behavioral outcomes were only assessed cross-sectionally. Thus, we cannot establish whether these behaviors are the consequence of certain parenting practices, or that they perhaps evoke these parenting practices. The same goes for eating style and activity style, which we regarded as relatively stable child characteristics, and therefore included as predictors of parenting practices. They could, however, also be influenced by parenting practices. Many of the previous studies in this research area have limited themselves to cross-sectional explorations, and there is a need for prospective research to establish causality [e.g., [7,21]]. It is reassuring, though, that the associations between two of the parenting practices (i.e., stimulation of healthy intake and restriction of sedentary time) and behavior were supported by the associations with later BMI development, pointing in the same direction. An additional strength is that the data in the current study were assessed prospectively, limiting the risk of recall bias and other problems inherent in retrospective research.

A major limitation of the current study is that all data, including dietary intake, activity behavior and anthropometrics, were self-reported by the parents, which may have led to bias. However, previous research has shown that parental reports of weight and height differed little from measured data [48]. An additional limitation is that the Cronbach's α values of some of our scales were relatively low. Although a Cronbach's α ≥.6 is generally considered acceptable [49], some authors advocate different cut-off points. Furthermore, caution is warranted when generalizing our results to the broader population of young Dutch children. Parents with an 'alternative', relatively healthy lifestyle were overrepresented in our sample, due to the choice of recruitment methods, i.e., recruiting some of the women from 'alternative lifestyle' circles [36]. The relatively healthy average lifestyle of our study sample is reflected in the children's relatively low mean BMI z-scores. However, secondary analyses showed that excluding the children who where underweight at age 5 did not change our findings. Moreover, all analyses were adjusted for recruitment channel. Finally, it may be noted that the reported effect sizes are small, indicating that the amount of variance in behavior and weight status explained by the parenting practices is limited. This may be partly attributable to the fact that parenting behavior is a concept that is hard to assess, and there is no consensus about the proper way to measure it. There are dozens of questionnaires assessing diet-related parenting practices, activity-related parenting practices, or both [e.g., [37,50-53]]. We feel quite confident, though, about the instruments adapted from the CFQ [37] for the current study, although the diet-related 'stimulation of healthy intake' scale and the 'Activity-related Parenting Questionnaire' were not previously validated. The fact that our adapted scales for 'stimulation of healthy intake' and 'restriction of sedentary time' predicted BMI change from age 5 to age 7 may be considered reassuring in this respect. Future research would benefit from a consensus about feasible and valid measurement methods.

## Conclusions

The current study showed that although most energy balance-related parenting practices were associated with desirable behaviors, there are also practices (e.g., restriction of sedentary time) that influence 5-year-old children's behavior and subsequent weight outcomes in a negative sense. Stimulating a child seems to be an effective practice to achieve both a healthy diet and a healthy activity pattern in children. However, the associations between several of the parenting practices and child behavior were found to depend on child characteristics, which calls for parenting that is tailored to each individual child.

## List of abbreviations

BMI: Body mass index; CFQ: Child Feeding Questionnaire; FFQ: Food Frequency Questionnaire; KOALA: Child, Parent and health: Lifestyle and Genetic constitution (in Dutch); PA: Physical activity.

## Competing interests

The authors declare that they have no competing interests.

## Authors' contributions

All authors made substantial contributions to the design of the study. JSG, AS, SIdV, RAG and CT were involved in the acquisition of the data. JSG analyzed and interpreted the data, and wrote draft versions of the manuscript. SPJK contributed to the interpretation of the data and the writing of the manuscript. All authors were involved in critically revising the manuscript, and have given their approval for the submitted manuscript.
